# *In-vitro* and *In-vivo* Evaluation of Silymarin Nanoliposomes against Isolated Methicillin-resistant *Staphylococcus aureus*

**Published:** 2015

**Authors:** Zohreh Faezizadeh, Amir Gharib, Masoud Godarzee

**Affiliations:** a *Department of Laboratory Sciences, Borujerd Branch, Islamic Azad University, Borujerd, Iran. *; b *Department of Biology, Borujerd Branch, Islamic Azad University, Borujerd, Iran.*

**Keywords:** Silymarin, Nanoliposome, Methicillin-resistant *Staphylococcus aureus*, Killing rate, *In-vitro*, *In-vivo*

## Abstract

*Staphylococcus* *aureus* is an opportunistic pathogen and remains a common cause of burn wound infections. Different studies have shown that entrapment of plant-derived compounds into liposomes could increase their anti-*Staphylococcus aureus *activity. Silymarin is the bioactive extract from the known plant *Silybum marianum L*. The objective of this study was to evaluate efficacy of silymarin in free and nanoliposomal forms against isolated methicillin-resistant *Staphylococcus aureus* (MRSA) strain. Silymarin-loaded nanoliposomes were prepared by extrusion method. The minimum inhibitory concentrations (MICs) of silymarin in free and nanoliposomal forms against MRSA were determined by broth dilution method. The killing rate of free and nanoliposomal forms of silymarin were analyzed. Ultimately, *in-vivo* therapeutic efficacy of nanoliposomes in burned mice infected by isolated MRSA was examined. The MICs of free and nanoliposomal forms of silymarin against isolated strain were 500 and 125 mg/L, respectively. The killing rate of silymarin-loaded nanoliposomes was higher than those of free silymarin. Topically treatment by silymarin in free and nanoliposomal forms resulted in almost 20 and 100% survival rates, respectively. The results suggest that silymarin-loaded nanoliposomes may provide a basis for future treatment of MRSA infections.

## Introduction

*Staphylococcus* *aureus* is an opportunistic bacterial pathogen causing skin infections in hospitals, especially burn units (1). Since *S.* *aureus* can rapidly disseminated from the burn wound sites into organs via the blood stream the clinical outcome in these patients can lead to sepsis which is often fatal (2). The major problem associated with *Staphylococcus* infection is resistant to penicillin, methicillin or other conventional antibiotics (1). Therefore, there is the compelling need to develop novel agents and possible strategies to overcome this resistance (3). Silymarin, a flavonolignan from 'milk thistle' (*Silybum marianum*) plant is composed mainly of six lignans including silychristin (SC), silydianin (SD), silybin A(SB_A_), silybin B (SB_B_), isosilybin A (ISB_A_), and isosilybin B (ISB_B_) and, possesses a range of biological and medical properties, including antioxidant, anti-cancer, anti-obesity, antiviral and antibacterial activities (4-6).

 Liposomes are spherical and colloidal vesicles can be used as a vehicle to drug delivery (7). These vehicles are composed of natural phospholipids, and may also contain other lipids such as cholesterol (8-11). It seems, liposome-entrapped antibiotics by increasing of bacterial membrane penetration, could reduce bacterial resistance (12, 13). Later studies demonstrated that encapsulation of plant-derived compound into liposomes markedly alters their pharmacokinetics, increasing half-lives and effectiveness (14, 15). Some of these derived such as epi-gallocatechin gallate (EGCG) and cyanidin have more antibacterial efficacy in liposomal form (12, 13). These efficacies, according to the literature, is related to type of plant-derived compound and interaction between their and liposomal membrane lipids and therefore have a major impact on therapeutic success (14, 15). The anti-MRSA effectiveness of silymarin-loaded nanoliposomes yet was not studied. The objective of this study was to prepare the silymarin-loaded nanoliposomes and evaluate its *in-vitro* and *in-vivo* antibacterial activity against isolated MRSA strain. 

## Experimental

*Materials*

Chemicals purchased from Sigma-Aldrich Chemical Company (St. Louis, USA) were silymarin, cholesterol and egg lecithin. Mueller-Hinton broth, dioxane, soybean casein digest agar (SCDA), chloroform, methanol was purchased from Merck (Darmstadt, Germany).

*Microorganism*

MRSA strain was isolated from clinical samples at Golestan Hospital (Ahvaz, Iran) and identified by using the reported method including tube coagulase test, slide coagulase test, latex agglutination test, Dnase and heat-stable nuclease tests, commercial biochemical tests, antimicrobial susceptibility test and PCR amplification and sequencing tests for some genes such as *mecA* (3, 16). This strain was inoculated onto blood agar plates and then incubated at 37 ˚C for 24 h and used for experimentation.

*Preparation of nanoliposomes*

Silymarin-loaded nanoliposomes were prepared using the method described previously (13). Briefly, the egg lecithin and cholesterol at the appropriate molar ratio (Table 1) were dissolved in chloroform and dried to a lipid film with a rotary evaporator (Brinkman, Toronto, Canada) under N2 flow and vacuum at 30 ˚C. The dried lipids were dispersed by agitation in silymarin solution and sonicated at 4 ˚C in ultrasonic bath (Braun-sonic 2000, Burlingame, USA). At finally, silymarin-loaded nanoliposomes were obtained by extruding of respective suspension using a polycarbonate membrane filter 100 nm-sized pores for 12 times and then for separation of excess free silymarin and larger lipid aggregation by ultracentrifugation (100000 g for 30 min). Control nanoliposomes were prepared similarly, but PBS (pH 7.4) was used instead of the silymarin solution.

**Table 1:** Lipid composition of silymarin-loaded nanoliposomes

**Lipids**	**Molar ratio of lipids (µmol/mL)**
Egg lecithin:Cholestrol	6:1

*Determination of encapsulation efficacy*

The content of silymarin in prepared nanoliposomes was determined by HPLC as previously described (17), following dissolution in 0.1% Triton X-100. To determination of silymarin, the 20 µL of nanoliposomal lysate was injected into the HPLC column. In the HPLC analysis, a C18 column (4 mm × 150 mm, 5 µm,Waters Co., Milford, USA) was used. The mobile phase was phosphoric acid (85%): methanol: water (0.5:46:64, v:v) at a flow rate of 1 mL/min. The detection was done at 288 nm. Each analysis cycle required 20 min. Silymarin corresponds to the sum of peak areas of SC, SD, SB_A_, SB_B_, ISB_A _and ISB_A_ concentrations. Then, the encapsulation efficiency defined as % encapsulation = (C_I_/C_T_) × 100, where C_I _– silymarin in nanoliposome, C_T _– total silymarin in the nanoliposome preparation, was used in calculation.

*Particle size, zeta-potential and polydispersity index determination*

Mean particle size, polydispersity index and zeta-potential of nanoliposomes was evaluated by Malvern zetasizer (Malvern instrument, Worcestershire, UK) apparatus, as described previously (18).

*Antimicrobial susceptibility testing*

The MICs of free and silymarin-loaded nanoliposomes for isolated MRSA strain were determined by the broth dilution technique as recommended by Clinical and Laboratory Standards Institute (CLSI) (19). Bacterial cell suspensions (~ 5×10^5^ cells/mL) were diluted in Mueller-Hinton broth and dispensed (100 μL) into a microtiter tray containing serial two-fold dilutions of silymarin and then incubated for 24 h at 37 ºC. The MIC was the lowest concentration of silymarin in free and nanoliposomal form that prevented visible bacterial growth and expressed in µg/mL. 

*Time-kill studies*

Time kill studies were preformed according to the method described previously (20). Briefly, 100 µL of MRSA suspension were resuspended in 10 mL of Mueller-Hinton broth and then incubated overnight at 37 °C, and adjusted to match the 0.5 McFarland turbidity. Subsequently, 100 µL of this standardized inoculums were added to separate culture tubes containing 1 mL of Mueller-Hinton broth with 1 mL free and nanoliposomal silymarin solutions at 1, 2 and 4 times the MIC and then incubated at 37 °C. The colony counts were performed at 0, 2, 4, 6, 8, 12 and 18 h and data were expressed as log colony forming unit per milliliter (CFU/mL).

*In-vivo study*

*In-vivo* therapeutic efficacy of silymarin-loaded nanoliposomes was tested by a described method (21), with some modification. In brief, forty male BALB/c mice (20-22 g) obtained from the National Institute of Pasture, Iran. Mice were handled according with the national guidelines of the laboratory animal and housed in separate cages and received water and food ad *libitum* (22). Animal care and protocols were performed and approved by the Institutional Animals Ethics Committee of Borujerd Branch, Islamic Azad University (Number: 202). After anesthetized with ketamine-xylazine mixture (150 mg/Kg, given intramuscularly), the mice back’s were shaved and a brass bar (10×10×100 mm) was heated in boiling water for 18 min and then applied on the shaved back of the mice for 50 seconds to burn induction. Then, 50 μL of the bacterial inoculums (containing 1×10^9^ CFU of total bacteria) was applied subcutaneously into the burned sites on the animal's back. The burned mice were divided into 4 groups. 

Prior to the treatment starting, the gel forms of the silymarin-loaded nanoliposomes, free silymarin, empty nanoliposomes and physiological saline were prepared according to the previously described method (23).

 All groups were treated topically as follows: Group 1 received silymarin-loaded nanoliposomes gel (150 mg/Kg/12h); groups 2 received free silymarin gel (150 mg/Kg/12); group 3 received empty nanoliposomes gel (150 mg/Kg/12h), and group 4 received physiological saline gel (1 mL/Kg/12h); for 12 days starting from the 4^rd^ day post infection. Three days after the last dose the surviving mice were anesthetized and sacrificed by cervical dislocation. Then, the skin, liver and spleen of animals were removed under sterile conditions and homogenized for 5 min in PBS (pH 7.4, 2 mL/g). The homogenates were then serially diluted and plated for growth in SCDA. At finally, the inoculated plates were incubated at 35 ºC for 24 h and the colony forming unit (CFU) was counted.

*Data analysis*

 All data were expressed as means ± SD. Statistical comparisons of killing rate study were performed by paired Student’s t-test, and p-value of less than 0.05 was considered significant. The data of survival rates were determined using ANOVA test.

## Results and Discussion

**Figure 1:**
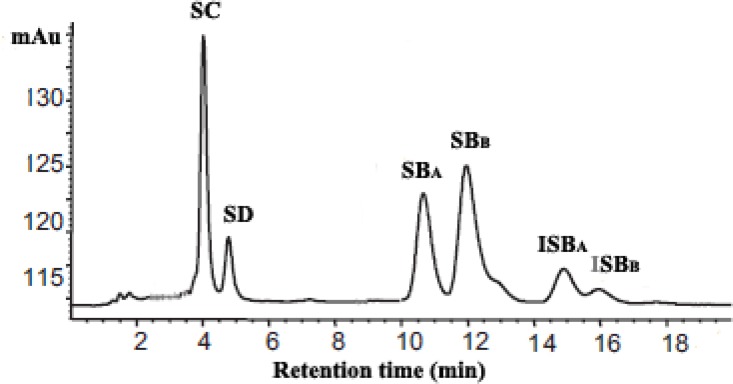
Chromatogram of silymarin analysis.

Recently, the use of plant-derived compounds to eliminate of MRSA has been widely investigated (24, 25). However, the main problems associated with application of some of these components are low water solubility and low bioavailability (12, 24). To overcome of these problems, the investigators were focused on entrapment of plant-derived compounds in drug carriers such as liposomes (13). Silymarin is a mixture of **flavonolignans** from the medical plant *Silybum marianum* (17). As shown in Figure 1, the silymarin content could be estimated from sum of its six lignans peak area. 

In this report, we evaluated the potential of incorporation of silymarin into nanoliposomes. Table 2 shows the zeta-potential, mean particle size, and polydispersity index of empty and silymarin-loaded nanoliposomes. Size homogeneity of empty and loaded nanoliposomes suggested that silymarin was entrapped into lipid bilayer, according to the previous studies (12, 15). Zeta-potential of nanoparticles revealed that prepared silymarin-loaded nanoliposomes have appropriate stability in aqueous dispersion (26). The results showed that silymarin can be encapsulated into nanoliposomes with high entrapment efficacy (83.00%±0.17). According to the previous studies, this phenomenon was probably due to the positive interaction between liposomal membrane lipids and loaded drugs that could be increase the encapsulation efficiacy of prepared liposomes (10, 11). It has been shown that EGCG with negative charge has high encapsulation efficacy in cationic liposomes (13). Therefore, it seems the weak forces such as the various known types of weak links between silymarin and liposomal lipids are effective factors in silymarin encapsulation.

**Table 2:** Particle size, zeta-potential and polydispersity index of empty and silymarin-loaded nanoliposomes

**Formulations **	**Mean particle size** **±SD** ** (nm)** **(n=10) **	**Zeta-potential** **±SD** ** (mV)** **(n=10) **	** Polydispersity index** **±SD** **(n=10) **
**Empty nanoliposomes**	93.20 ±0.11	-1.70±0.52	0.31±0.07
**Silymarin-loaded nanoliposomes**	95.50 ±0.25	-1.40 ±0.31	0.31±0.02

The MIC values of silymarin in either free or nanoliposomal form for isolated MRSA were shown in Table 3. The MIC of silymarin-loaded nanoliposomes was lower than those of free form, respectively. Our results suggest that entrapped of silymarin in nanoliposomal form enhanced the anti-MRSA activity of its compared to free silymarin. The data from this study is according to previous report, indicating the encapsulated of oleic acid (a fatty acid found naturally in many plant) in liposomes could eliminate MRSA as well (25). Several hypotheses, including non-sensitivity of plant-derived compounds to bacterial enzymes and increased penetration of nanoliposomes into bacteria cells may explain the effectiveness of these formulations (25, 27).

**Table 3:** *In* *-* *vitro* antimicrobial activities of free and nanoliposomal forms of silymarin against isolated MRSA

	**Minimum inhibitory concentration (mg/L)**	
Drugs	**Free silymarin**	**Silymarin-loaded nanoliposomes**
Microorganism Isolated MRSA strain	500	125

**Figure 2:**
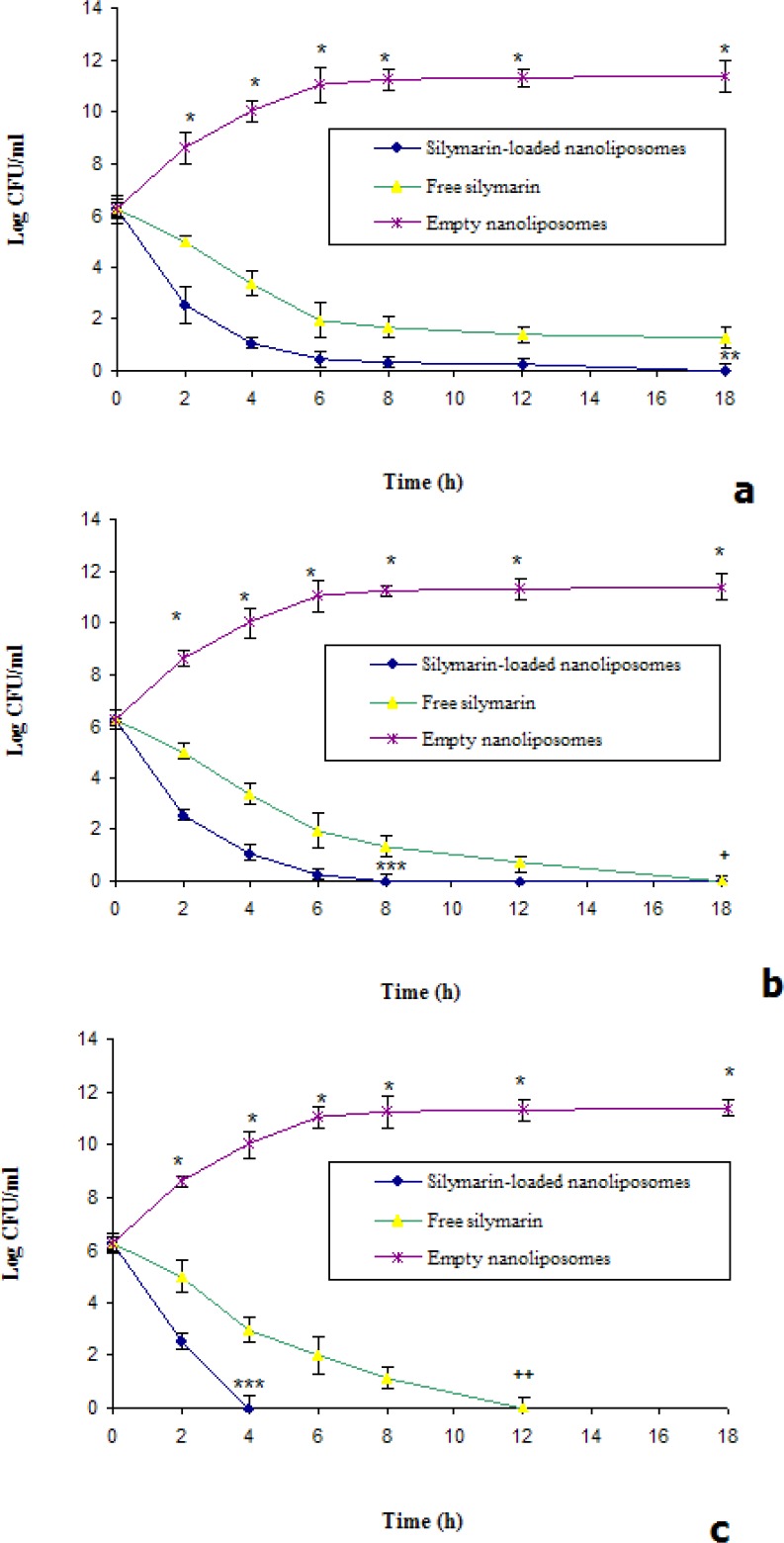
Killing curves for isolated strain *S. aureus* was exposed to various concentrations (a=1×MIC, b=2×MIC and c=4×MIC) of silymarin in free and nanoliposomal forms. *Significant difference between killing rate of empty nanoliposomes versus free and silymarin-loaded nanoliposomes (p<0.01), **Significant difference between killing rate of silymarin-loaded nanoliposomes versus free silymarin (p<0.05), ***Significant difference between killing rate of silymarin-loaded nanoliposomes versus free silymarin (p<0.01), ^+^Significant difference between killing rate of free silymarin and empty nanoliposomes (p<0.05).^ ++^Significant difference between killing rate of free silymarin and empty nanoliposomes (p<0.01).

The killing curves of silymarin in free and encapsulated form at 1, 2 and 4 times the MICs were shown in Figure 2. In all conditions, silymarin-loaded nanoliposomes were more effective on reduced bacterial counts compared to free silymarin (Figure 2). At once of MIC only silymarin encapsulated in nanoliposomes could eliminate of MRSA after 18 h (Figure 2a). At twice of MIC, the encapsulated and free silymarin could eradicate the bacteria after 8 and 18 h, respectively (Figure 2b). At four times of MIC, silymarin-loaded nanoliposomes could eliminate the bacteria after 4 h (Figure 2c). Our data are accordance with previous study, which reported that significantly higher killing rates of MRSA with liposomal EGCG or oleic acid were occurred (13, 25). So, we hypothesized that interaction between the outer membrane lipopolysaccharides of S. aureus and nanoliposomes could enhance the silymarin effectiveness.

*In-vivo* testing showed that the treatment of the skin infected mice with silymarin-loaded nanoliposomes could reduce significantly CFU values in evaluated organs, especially in spleen and liver (Table 4). Moreover, in other organ such as kidneys and brains of treated mice with silymarin-loaded nanoliposomes the growth of MRSA was not observed (data not shown). It was found that mortality of animals as control (without administration of silymarin) was 100% after 10 days, whereas mice treated with silymarin in free and nanoliposomal form showed the increase in survival rate of 20 and 100%, respectively. Wounds, especially burn wounds and other exposed tissues are particularly susceptible to bacterial contamination and infections (28, 29). According to previous report, the potential mortality from burn wound infections, even after aggressive antibiotics therapies, may approach 50% (30). Treatment of mice with silymarin-loaded nanoliposomes resulted in 100% survival rate and in almost complete eradication of the MRSA from the skin and spleen of infected animals. These results may be due to the optimal antibacterial delivery that reported by several investigators (31, 32). When liposomes containing antibacterial compounds are applied, they may interact with the cell membranes of bacteria and this condition can cause increased drug concentration around the bacteria (12, 25).

**Table 4:** The survival rate of infected mice and colony-forming units (CFUs) of isolated MRSA in different organs

** Treatment**	**Tissue/Organ**	**Log CFU/Gram tissue**	**Percentage of survival mice (n=10)**
Control without drug administration (Received physiological saline, 150 mg Kg^-1^, Topically)	Liver Skin Spleen	2.27 ± 0.40 2.57 ± 0.21 2.13 ± 0.63	None survived
Empty nanoliposomes (150 mg Kg^-1^, Topically)	Liver Skin Spleen	2.11 ± 0.41 2.56 ± 0.84 2.41 ± 0.62	None survived
Free silymarin (150 mg Kg^-1^, Topically)	Liver Skin Spleen	1.12 ± 0.43 1.75 ± 0.65 1.47 ± 0.31	20
Silymarin-loaded nanoliposomes (150 mg Kg^-1^, Topically)	Liver Skin Spleen	Nil* 0.43 ± 0.12** Nil*	100

The results are expressed as Mean ±Standard error of mean from three separate experiments. Analysis of variance of one-way classification between the treatment means was heterogeneous and the t-test values (two-tailed) were significant,

* *p* <0.001 and

** *p* < 0.05.

In conclusion, *in-vitro* and *in-vivo* testing of silymarin-loaded nanoliposomes indicated that this formulation has strong protective functions against MRSA and would be a good choice for treatment of patients with MRSA infections.
